# Stable Isotope Labeling-Based Nontargeted Strategy for Characterization of the In Vitro Metabolic Profile of a Novel Doping BPC-157 in Doping Control by UHPLC-HRMS

**DOI:** 10.3390/molecules28217345

**Published:** 2023-10-30

**Authors:** Tian Tian, Jing Jing, Yuanyuan Li, Yang Wang, Xiaojun Deng, Yuanhong Shan

**Affiliations:** Shanghai Anti-Doping Laboratory, Shanghai University of Sport, Shanghai 200438, China; tiantian@sus.edu.cn (T.T.); jingjing@sus.edu.cn (J.J.);

**Keywords:** stable isotope labeling, BPC-157, metabolic profile, doping control, UHPLC-HRMS

## Abstract

Traditional strategies for the metabolic profiling of doping are limited by the unpredictable metabolic pathways and the numerous proportions of background and chemical noise that lead to inadequate metabolism knowledge, thereby affecting the selection of optimal detection targets. Thus, a stable isotope labeling-based nontargeted strategy combined with ultra-high-performance liquid chromatography–high-resolution mass spectrometry (UHPLC-HRMS) was first proposed for the effective and rapid metabolism analysis of small-molecule doping agents and demonstrated via its application to a novel doping BPC-157. Using ^13^C/^15^N-labeled BPC-157, a complete workflow including automatic ^13^C_0_,^15^N_0_-^13^C_6_,^15^N_2_ *m*/*z* pair picking based on the characteristic behaviors of isotope pairs was constructed, and one metabolite produced by a novel metabolic pathway plus eight metabolites produced by the conventional amide-bond breaking metabolic pathway were successfully discovered from two incubation models. Furthermore, a specific method for the detection of BPC-157 and the five main metabolites in human urine was developed and validated with satisfactory detection limits (0.01~0.11 ng/mL) and excellent quantitative ability (linearity: 0.02~50 ng/mL with R^2^ > 0.999; relative error (RE)% < 10% and relative standard deviation (RSD)% < 5%; recovery > 90%). The novel metabolic pathway and the in vitro metabolic profile could provide new insights into the biotransformation of BPC-157 and improved targets for doping control.

## 1. Introduction

The continuing progress of modern anti-doping analytics has allowed for great achievements in the last few years, enabling the detection of infinitesimal amounts of prohibited substances and thereby effectively reducing the occurrence of doping events [1]. However, new doping agents keep being discovered and used in professional sports competitions in an attempt to enhance performance [1,2]. For example, BPC-157, a novel emerging peptide-derived drug (GEPPPGKPADDAGLV), has been classified as S0. Non-approved substances was added to the 2022 Prohibited List by the World Anti-Doping Agency (WADA) due to its potential to enhance athlete performance [3]. As a fragment of protein BPC (body-protecting compound, 40 kDa), BPC-157 (also called PL 14736, PL-10, and bepecin) is thought to be sufficient and responsible for the protective and physiological effects [4]. Pharmacological studies, as well as non-scientific online testimonials, both suggest that this peptide aids in muscle, tendon, and ligament healing, promoting cytoprotection and wound healing [5,6,7,8]. Thus, despite proper clinical trials in human subjects having not yet been performed to substantiate the healing claims and potentially harmful effects of BPC-157, it is being used by athletes looking to gain a competitive edge [9]. For this reason, it is important to have a deep awareness of biotransformation for such new prohibited substances and implement it into routine doping screening, considering the health risks to athletes and the fairness of competitions. To date, limited information is available on the metabolism of this substance, prompting the need for further research and analysis.

Logically, the most straightforward and effective path for the metabolic study is the human administration of the target. However, this is generally difficult to conduct due to ethical constraints and safety risks, especially for new doping agents, since the majority may not be approved for marketing [10,11]. In response to this limitation, proper in vitro models have been sought out for the better prediction of doping metabolites, such as human serum/plasma, liver, or kidney microsomes and the S9 fraction, and recombinant proteases [1]. Among them, studies with subcellular fractions, such as microsomes and the S9 fraction, usually provide a high number of metabolites and are useful to quickly generate many potential metabolites and obtain retention times (RTs) and mass spectra to be matched with in vivo samples. Combined with advanced analytical techniques, especially UHPLC-HRMS-based approaches, remarkable progress has been made in the investigation of doping metabolism based on in vitro experiments [12,13]. However, some issues remain challenging. The conventional strategy is based on the drug structure combined with commercial software or researchers’ experience for metabolite prediction [14,15], which may lead to inadequate metabolic knowledge and missing metabolite information because the metabolic pathways cannot be fully foreseen, especially affecting the finding of potential long-term metabolic targets [15,16]. In addition, the incubation systems are complicated, and a large amount of mass spectral information contains numerous proportions of background and chemical noise, which prevents the effective and rapid screening of targets.

Recently, a promising approach referred to as stable isotope labeling has attracted extensive interest in the fields of metabolomics, single-cell omics, disease biomarker exploring, and natural product screening, etc., and was proved to be efficient in the screening of endogenous or exogenous metabolites [17,18,19,20]. Based on the same physicochemical properties and characteristic mass difference of isotope pairs, combined with HRMS, it is specific and rapid for nontargeted screening and enables the unbiased identification of numerous metabolites. For example, upon isotope-coded derivatization with a pair of labeling reagents, HBP-d (0) and HBP-d (5), characteristic neutral fragments of 79 Da and 84 Da were generated for the aldehyde analytes [19]. In combination with the unique isotopic doubles (Δ*m*/*z* = 5 Da) and dual neutral loss scanning (dNLS), it allowed the nontargeted profiling and identification of endogenous aldehydes even amidst noisy data. Accordingly, a generic screening strategy was developed by Huang et al. [19], and its application to the screening of cinnamon extracts led to the finding of 61 possible natural aldehydes and guided the discovery of 10 previously undetected congeners in this medicinal plant. In terms of metabolomics analysis, using newly designed stable isotope-labeled reagents ([d(0)]-/[d(3)]-/[d(6)]-DMMIC), Liu et al. [21] achieved rapid nontargeted screening of amine submetabolomes in human esophageal tissues based on the formed set of molecular ions with an increase of 3.02 *m*/*z* and characteristic fragment ions with *m*/*z* 204.1: 207.1: 210.1. Through this technique’s application in the analysis of metabolic differences between the carcinoma and paracarcinoma tissues of esophageal squamous cell carcinoma, 13 amine metabolites were discovered with the highest potential differential. Furthermore, the absence and presence of isotopic labels in reaction products can trace reaction pathways to reveal the reaction mechanisms. Based on ^13^C stable isotope labeling in combination with HPLC-HRMS, Geng et al. [22] discovered the binding products of quinone–quinone that are not derived from either nucleophilic reactions or redox reactions, providing a new perspective for quinone metabolic pathway in foods. In the field of anti-doping, stable isotope labeling-based strategies have also been attempted for metabolite screening. Thevis et al. [13,23] developed an isotope-labeled reporter ion screening strategy for prohibited protein and large peptide substances and demonstrated its feasibility by applying it to the metabolite identification of HGH, IGF-1, etc. In their study, the feature of commonly appearing immonium ions generated by the internal dissociation of single amino acids from the peptide backbone as “flags” for metabolites was exploited. The signals of metabolites can be obtained by extracting labeled immonium ions from the AIF data, while the exact intact mass of the analytes may be hard to determine. In addition, for small-molecule doping agents, characteristic diagnostic ions similar to large peptides are not easily generated. Thus, a proper isotope labeling strategy for the metabolism analysis of small-molecule doping agents, which are the major components of the WADA prohibited list, needs to be developed.

In the present study, a stable isotope labeling-based nontargeted strategy combined with UHPLC-HRMS was first proposed for metabolism analysis of small-molecule doping agents to realize the rapid and effective screening of targets and demonstrated via its application to the in vitro metabolism of a novel peptide doping BPC-157. Although Cox et al. [9] reported the in vitro plasma metabolism of BPC-157 and found several degradation metabolites, more in-depth studies with different in vitro incubation models need to be performed to further elucidate the biotransformation in organisms due to the diversity of administration routes. Using ^13^C/^15^N-labeled BPC-157, based on the similar chromatographic behavior and fixed mass differences of isotope pairs, a complete workflow that includes automatic ^13^C_0_,^15^N_0_-^13^C_6_,^15^N_2_ *m*/*z* pair picking was constructed to mine potential metabolites in the human liver microsomes and human skin S9 incubation systems. As a result, extensive metabolism was observed in the two incubation systems with an obvious difference, and one metabolite produced from a novel metabolic pathway plus eight metabolites produced from the conventional amide-bond breaking metabolic pathway were discovered in total, providing six more metabolites than a previous study [9]. The five main metabolites were identified with the aid of synthetic reference materials (RMs). Furthermore, a sensitive and specific method for qualitative and quantitative analysis of BPC-157 and the five main metabolites in human urine was first developed and validated systematically with satisfactory results. The novel metabolic pathway and the metabolic profile in the two incubation models could provide new insights into the metabolic process of BPC-157 in organisms and improved analytical targets for doping control.

## 2. Results and Discussion

### 2.1. Stable Isotope Labeling-Based Nontargeted Strategy for the Metabolism Analysis

Traditional metabolite screening technologies are limited by unpredictable metabolic pathways and the numerous proportions of background in mass spectral information that lead to inadequate metabolism knowledge. Within this study, a stable isotope labeling-based strategy combined with UHPLC-HRMS was developed for the metabolic profiling of small-molecule doping agents for the first time to achieve the effective and rapid screening of targets. Differentiated from the method reported by Thevis et al. [13], the strategy established here was based on differences in the mass spectrometric features (fixed mass differences in ions and similar cleavage behaviors) and the chromatographic features (highly similar retention times and peak shapes) between the light and heavy labels for metabolite mining, which was more suitable for small-molecule doping agents.

**Figure 1:**
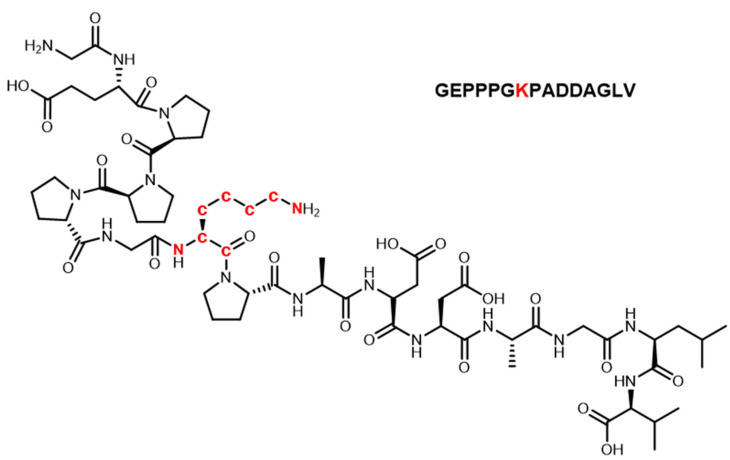
Primary structure of BPC-157 (isotope-labeled atoms were shown in red).

**Figure 2:**
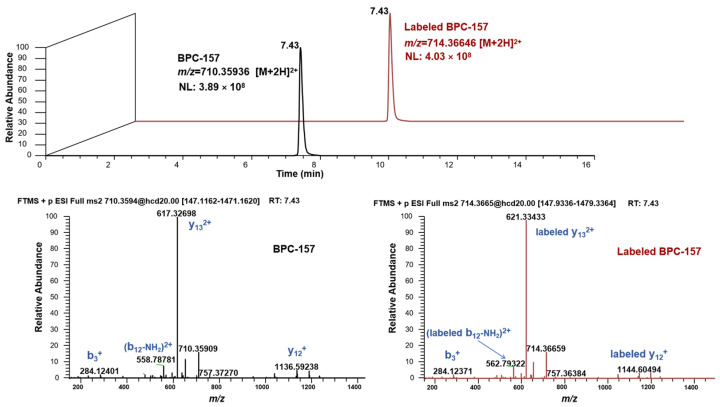
Chromatographic and mass spectrometry behavior of BPC-157 and labeled BPC-157.

The labeling site and number of heavy isotopes were key considerations in developing the strategy. For the metabolic analysis of small-molecule doping agents, the labeling sites are preferred to groups that are not easily involved in the metabolic process, such as the benzene ring, alicyclic ring, and heterocyclic ring, so that the metabolite ion pairs with isotopic characteristics can be retained to the maximum extent. According to previous studies [24,25,26], peptides are usually metabolized through the degradation of amino acids at both ends to form metabolites in the body. Thus, in this study, the lysine in the middle of the peptide sequence was selected to be labeled with heavy isotopes to avoid the loss of metabolite information as much as possible, although this cannot be completely avoided. The number of heavy isotopes labeled for small-molecule doping is usually no less than three so that it can form a significant mass difference. For peptides, the labeled number needs to be greater because such substances tend to form multi-charged ions (mainly in double charge) due to the carboxyl and amino groups of amino acid residues in the structure. Accordingly, the carbon and nitrogen atoms on the lysine of BPC-157 were all labeled with ^13^C and ^15^N, given that significant mass differences can be formed (Δm = 8.01420 Da) (Figure 1). In addition, ^13^C and ^15^N are more stable and not as easily metabolized as deuterium. Under the current LC-MS conditions, there are several notable characteristics in the chromatographic and mass spectrometric behaviors between the labeled and unlabeled BPC-157, as shown in Figure 2: (1) they have RT differences within 0.05 min; (2) they share similar peak shapes and signal intensities at the same concentration; (3) they share the same kinds of additive ions that are detected in the major form of +2 charge, and the *m*/*z* values show an exact difference of 4.00710 ± 0.003; and (4) they share the same cleavage pathway in the secondary mass spectrum. Thus, it was speculated that these characteristics will also be present in the labeled and unlabeled metabolites. Based on this, a custom program was edited to automatically pick ^13^C_0_,^15^N_0_-^13^C_6_,^15^N_2_ ion pairs with *m*/*z* values separated by exactly 4.00710 ± 0.003 and ΔRT within 0.05 min. Given the high resolution of the Orbitrap mass spectrometer, the mass window for screening ion pairs could be set at the millidalton level, greatly reducing the false positive rate. In addition, based on similar peak shapes and signal intensities, the false positive rate was further reduced. Finally, elemental composition analysis and targeted MS/MS were performed to further confirm the suspected metabolites and provide structural information.

**Figure 3:**
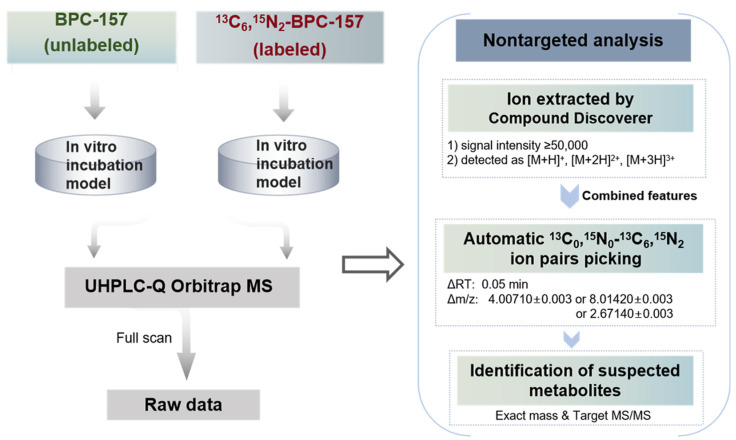
Data analysis workflow for nontargeted screening of all potential metabolites based on stable isotope labeling.

The data processing workflow for the nontargeted screening of potential metabolites is illustrated in Figure 3. Data acquisition of the ^13^C_0_,^15^N_0_/^13^C_6_,^15^N_2_-BPC-157-incubated samples was performed with full scan mode, and the raw data were ion-extracted with the following parameters using Compound Discoverer software 3.1: (1) ion peak signal intensity ≥50,000; and (2) ions detected as [M+H]^+^, [M+2H]^2+^, and [M+3H]^3+^. Although BPC-157 and labeled BPC-157 were mainly detected in the form of +2 charge, +1 and +3 charges were also set up to extract metabolite signals that may exist primarily in these forms. The resulting features were exported to an Excel file and combined for subsequent isotope ion pair picking using the custom program. Two important criteria were set in the automatic ion pair picking for the suspected metabolites. The first criterion was that the ion pairs with *m*/*z* values should be separated by exactly 4.00710 ± 0.003 or 8.01420 ± 0.003 or 2.67140 ± 0.003. Second, the ion pairs should have an RT difference of less than or equal to (≤) 0.05 min. Further analysis was performed for the candidate ion pairs as follows: (1) eliminating repeat features and ion pairs detected in the blank control samples; and (2) ensuring that the ion pairs have similar peak shapes and intensities. To confirm the remaining suspected metabolites, targeted LC-MS/MS acquisition was performed to ensure that there were metabolites produced in the same metabolic pathway in the ^13^C_0_,^15^N_0_/^13^C_6_,^15^N_2_-BPC-157-incubated samples and verify the structural information. Finally, based on the exact *m*/*z* values provided by HRMS and the product ion information, the elemental composition of the metabolites was determined. The entire screening process is based on a nontargeted analysis method, including nontargeted data acquisition with full scan mode, nontargeted ion extraction using molecular features, and automatic isotope ion pair picking, which can minimize the discriminatory treatment of metabolite information and enable comprehensive data mining.

### 2.2. Metabolic Profile of BPC-157 in Two Kinds of In Vitro Incubation Models

**Figure 4:**
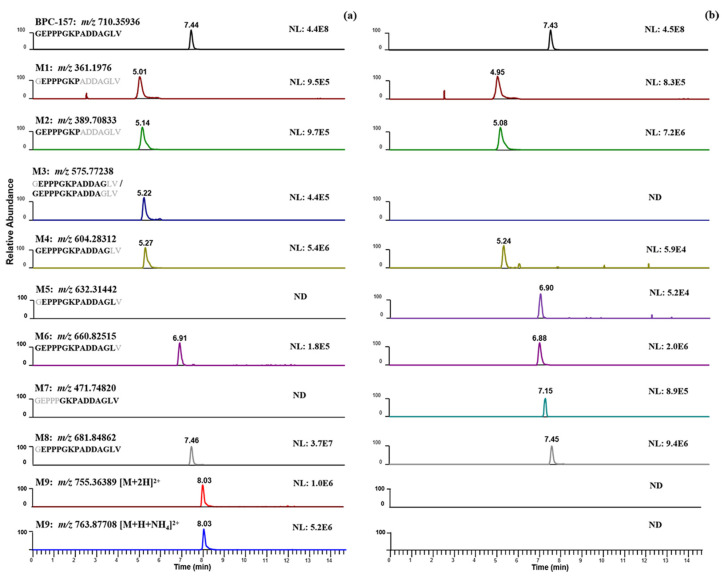
Candidate metabolites of BPC-157 detected in two in vitro incubation systems. (**a**) Human liver microsome incubation system; (**b**) human skin S9 incubation system.

As described in the literature, BPC-157 acts systemically in the body, which means that it can render a certain amount of benefit in whichever part of the body needs healing via subcutaneous/intramuscular injection or oral administration. Accordingly, the human skin S9 fraction and human liver microsomes were selected as in vitro incubation models in this study to simulate different drug delivery pathways of BPC-157 in humans for the first time. After incubation at 37 °C for 2 h, following a simple sample preparation of protein precipitation with acetonitrile, the metabolite information was retained to a large extent for nontargeted screening. Analyzed with the presented strategy (Figure 3), extensive metabolism was observed in the two incubation systems, but with an obvious difference. The different protein concentrations in the two systems may account for the different metabolic activity. As shown in Figure 4, five candidate metabolites were detected in both incubation systems with a different content, and four candidate metabolites were present in only one of them. For example, BPC-157 (2–14) free acid (M5) and BPC-157 (6–15) free acid (M7) were only detected in the human skin S9 incubation system, while *m*/*z* 575.77238 (M3), which may be BPC-157 (2–13) free acid or BPC-157 (1–12) free acid, *m*/*z* 755.36389 (M9, [M+2H]^2+^), and *m*/*z* 763.87708 (M9, [M+H+NH_4_]^2+^) were only detected in the human liver microsome incubation system. Similarly, for the labeled BPC-157, metabolites produced by the same metabolic pathways were also detected with an increase of 4.00710 ± 0.003 in the *m*/*z* (+2 charge state). Since it cannot be completely ruled out that the metabolism will not occur on the labeled lysine, metabolites associated with lysine degradation may be lost, although a previous study suggested that the lysine is not involved in the metabolic processes of BPC-157 [9]. Nevertheless, no possible metabolite signals formed by lysine cleavage were found via manual extraction in both incubation systems.

**Figure 5:**
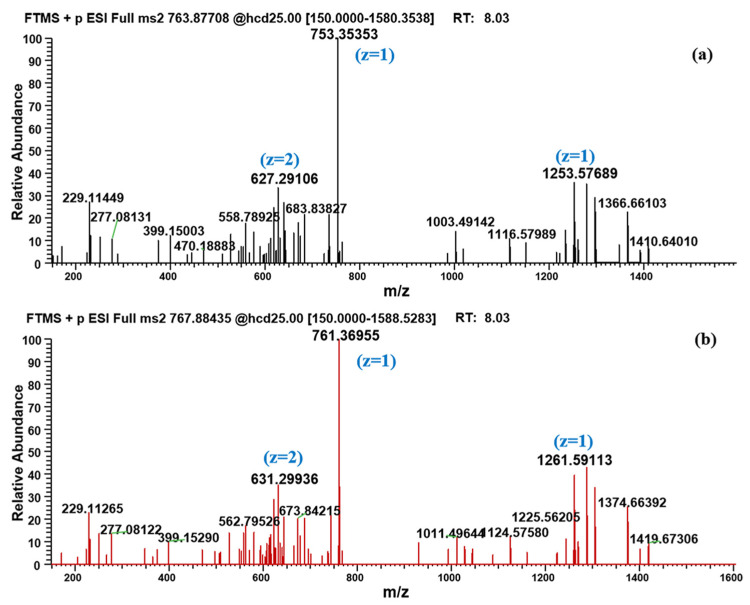
MS/MS spectrum of (**a**) *m*/*z 763.87708* (M9, [M+H+NH_4_]^2+^]) and (**b**) *m*/*z 767.88435* (labeled M9, [M+H+NH_4_]^2+^]).

It is worth noting that in addition to the traditional amide-bond-breaking metabolic pathway that leads to M1-M8, a temporarily unknown and novel metabolic pathway for BPC-157 was discovered through the proposed strategy. The hereby produced metabolite M9 has a larger molecular weight than the parent, which is unlikely to be formed by the degradation of the peptide chain alone. The metabolite existed primarily as an adduct ion peak of [M+H+NH_4_]^2+^ (763.87708) (Figure 4), probably because it is a peptide derivative. Another possibility has also been considered—that is, *m*/*z* 755.36389 was a deamination peak formed by the in-source fragmentation of *m*/*z* 763.87708. However, no fragment with *m*/*z* 755.36389 was observed in the MS/MS spectrum of *m*/*z* 763.87708, meaning that no convincing evidence could be found for this possibility. According to the MS/MS spectrum (Figure 5), characteristic fragments of *m*/*z* 753.35353, *m*/*z* 1253.57689, and *m*/*z* 627.29106 (+2 charge state of ion *m*/*z* 1253.57485) were generated for M9 under proper collision energy (CE). Similarly, fragments with an increase of 8.01420 ± 0.003 or 4.00710 ± 0.003 in *m*/*z* were detected in the MS/MS spectrum of labeled M9, such as *m*/*z* 761.36955, *m*/*z* 1261.59113, and *m*/*z* 631.29936. Then, based on the exact *m*/*z* provided by Orbitrap, attempts were made to speculate on the elemental composition of the metabolite. However, a number of molecular formulas were obtained because of the large molecular weight, but simple types of elements, and the final molecular formula has not been determined. The five possible molecular formulas are listed in Table 1, based on a comprehensive analysis of the number of elements, degree of unsaturation, and mass errors. The obtained *m*/*z* information can provide a reference for further research in organisms. No signal of this metabolite was detected in the human skin S9 incubated samples, suggesting that there may be some metabolic differences between the oral and injection administration of BPC-157. To the best of our knowledge, the generation of small peptide metabolites has been considered to originate from the cleavage of amide-bond [1], and the metabolite M9 undoubtedly provides new insights into the biotransformation of not only BPC-157 but also other small peptides.

**Figure 6:**
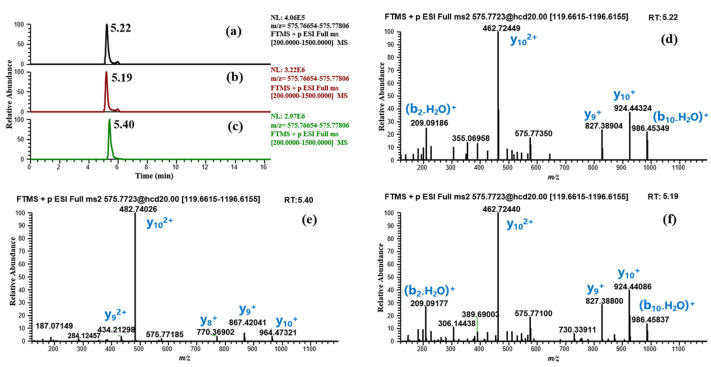
Confirmation of M3 based on synthetic RMs. (**a**) Extracted ion chromatogram (EIC) of *m*/*z* 575.77238 (M3) in human liver microsomes incubated sample; (**b**) EIC of the RM BPC-157 (2–13) free acid; (**c**) EIC of the RM BPC-157 (1–12) free acid; (**d**) MS/MS spectrum of *m*/*z* 575.77238 (M3); (**e**) MS/MS spectrum of the RM BPC-157 (1–12) free acid; (**f**) MS/MS spectrum of the RM BPC-157 (2–13) free acid.

**Figure 7:**
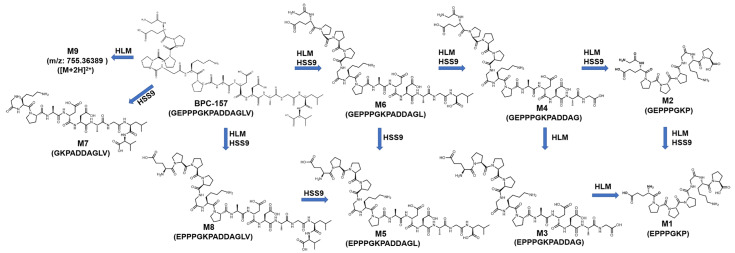
Metabolic profile of BPC-157 in two kinds of in vitro incubation models (HLM: human liver microsomes; HSS9: human skin S9).

Subsequently, based on results obtained from the two incubation models, RMs were synthesized for the confirmation of the major candidate metabolites. According to the detection results, four candidate metabolites were consistent with the RMs in RT, precursor ion, and MS/MS fragmentation, confirming our hypothesis (Table 2 (M1, M2, M4, M8)). For *m*/*z* 575.77238 (M3), the RMs of BPC-157 (2–13) free acid and BPC-157 (1–12) free acid were both synthesized and detected by LC-HRMS. Obvious differences were observed in the RT and MS/MS spectrum between the two, while the former matched the metabolite M3 (Figure 6). According to Cox’s report [9], *m*/*z* 575.77238 was detected as the co-elution of metabolites BPC-157 (2–13) free acid and BPC-157 (1–12) free acid. However, the RM detection results here show that the two substances can be separated under appropriate chromatographic conditions, and BPC-157 tends to cleave the first amino acid at the C-terminus and the last two amino acids at the N-terminus, resulting in BPC-157 (2–13) free acid (Figure 6). Altogether, nine different metabolic products were identified by employing the combination of human skin S9 and human liver microsomes-based in vitro metabolism and evaluation of the HRMS data with the presented strategy (Table 2, Figure 7), providing six more metabolites than a previous in vitro plasma metabolism study [9], potentially representing new target metabolites for the doping detection of BPC-157 administration. Among them, BPC-157 (2–15) free acid (M8) was the most abundant metabolite in both incubation systems, which is consistent with the previous report [9], while the relative response of other metabolites showed different ratios in the range of 0.5% ~ 77% (Table 2). Hence, differences in the proportion of metabolites excreted in different body fluids, such as the urine and blood, should be noted. In terms of the metabolic pathway of amide-bond breakage, BPC-157 is more likely to be degraded from the C-terminus to form a series of truncated peptide metabolites (Figure 7). The polarity of these truncated peptide metabolites gradually increased with the degradation of amino acids at the C-terminus—that is, the RT gradually moved forward (Figure 4), which may be attributed to the fact that amino acids at the N-terminus are mostly hydrophilic amino acids (e.g., lysine, glutamic acid, and proline), and amino acids at the C-terminus are mostly hydrophobic amino acids (e.g., valine, leucine, and alanine). As a previous study reported [1], the size of small peptides (<15–20 amino acids residues) is far below the threshold for glomerular filtration, so they generally undergo fast renal elimination followed by excretion in urine [27], making this the preferred matrix for doping testing. Thus, these metabolites are likely to be detected in urine due to their small size and high polarity. In addition, the metabolic pathway of degrading the first amino acid at the N-terminus was fully validated in the in vitro metabolic process of BPC-157, as M8 and BPC-157, M1 and M2, M3, and M4, and M5 and M6 all differ by one glycine (the first amino acid at the N-terminus) (Figure 7). In summary, the in vitro metabolic profile of human skin S9 and human liver microsomes provided a further reference for the in vivo biotransformation of BPC-157.

**Figure 8:**
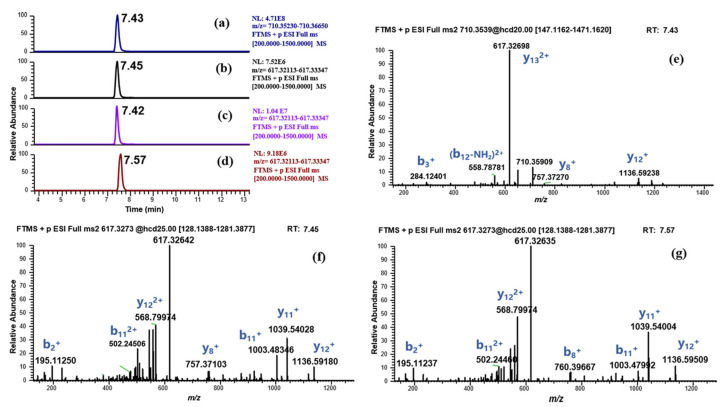
Identification of the suspected metabolite (BPC-157 (3–15) free acid, *m*/*z* 617.32733). (**a**) EIC of BPC-157 in human liver microsomes incubated sample; (**b**) EIC of *m*/*z* 617.32733 in human liver microsomes incubated sample; (**c**) EIC of *m*/*z* 617.32733 in enzyme blank sample; (**d**) EIC of the RM BPC-157 (3–15) free acid; (**e**) MS/MS spectrum of the RM BPC-157; (**f**) MS/MS spectrum of *m*/*z* 617.32733 in human liver microsomes incubated sample; (**g**) MS/MS spectrum of the RM BPC-157 (3–15) free acid.

Interestingly, we also observed the signal of another suspected metabolite (BPC-157 (3–15) free acid, *m*/*z* 617.32733) in both incubation systems with high abundance (Figure 8, the human liver microsomes incubation system was taken as an example), which has been reported as a metabolite of BPC-157 in Cox’s study [9]. However, some issues were noted. The metabolite shared the same RT and peak shape with the parent and also existed in the enzyme blank sample with higher intensity (Figure 8). In addition, a major product ion (y13) produced by the MS/MS fragmentation of the parent showed the same *m*/*z* (617.32733) and charge (+2) with the suspected metabolite (Figure 8e). Thus, it was considered that BPC-157 was prone to in-source fragmentation, and the suspected metabolite was speculated to be a fragment (y13) of BPC-157. As the amount of the parent is reduced due to metabolic reactions, the amount of this fragment is also reduced, accounting for the lower signal of this suspected metabolite in the incubation system than that in the enzyme blank sample. Subsequent detection results of RM BPC-157 (3–15) free acid confirmed this conjecture. The suspected metabolite has the same MS/MS fragment as the RM BPC-157 (3–15) free acid (Figure 8f,g), which means that the two have the same amino acid sequence, but the RT proved that they are not the same substance. The RTs for the suspected metabolite and RM BPC-157 (3–15) free acid were 7.45 min and 7.57 min, respectively (Figure 8b,d), which exceeded the RT-based qualitative criteria (0.1 min) for small molecules. In fact, in addition to the major fragment, several other minor in-source fragments of BPC-157 that may be mistaken for degradation metabolites were also found. Therefore, MS/MS experiments, as well as enzyme blank sample analysis, should be carefully performed to eliminate false positive results, given the risk of misidentification due to in-source fragmentation.

### 2.3. Method Development and Validation for Doping Control of BPC-157

For the purpose of anti-doping screening, a sensitive and specific method was established and validated for BPC-157 and the major metabolites in human urine in this study. Mixed-mode weak cation exchange solid phase extraction (WCX-SPE) offers both cation exchange and hydrophobic interactions that are frequently used for the detection of small peptides in urine samples. Thus, based on our previously developed small peptide detection method [28], minor optimizations were made to the WCX-SPE procedure and chromatographic conditions to make them more suitable for the detection of BPC-157 and the metabolites. The validation results are described below and summarized in Table 3, fulfilling the requirements of WADA for routine analysis of small peptides [29]. In addition, the results of validation parameters such as linearity, accuracy, precision, recovery, and matrix effects also demonstrated the potential of the method for quantitative analysis.

#### 2.3.1. Selectivity and Limit of Detection (LOD)

As the parallel reaction monitoring (PRM) mode with high resolution was used for data acquisition, the signal interference in urine was eliminated to a large extent. Therefore, no obvious interference was observed at the expected RTs in the twenty blank urine samples, illustrating that the method was selective, and the false positive rate of this method can be controlled effectively. Under optimized conditions, the method has satisfactory detection sensitivity that is well below the minimum required performance level (MRPL) designated for peptides by WADA (2 ng/mL) [29]. As shown in Table 3, the LOD for BPC-157 in the present study was 0.01 ng/mL, which was significantly lower than that in the previous study (0.1 ng/mL) [9]. In addition, this study involved the first detection of the metabolites of BPC-157 with relatively low LODs (0.03~0.11 ng/mL).

#### 2.3.2. Linearity, Repeatability, and Accuracy

The linearity of the method was investigated by preparing a series of spiked urine samples ranging from 0.02 to 50 ng/mL (0.02, 0.05, 0.1, 0.2, 0.5, 1, 2, 5, 10, 20, and 50 ng/mL) for all targets. Calculated with an external standard method, BPC-157 and BPC-157 (1–8) free acid (M2) showed good linearity in the range of 0.02~50 ng/mL with R^2^ > 0.999; BPC-157 (2–8) free acid (M1), BPC-157 (2–13) free acid (M3), and BPC-157 (1–13) free acid (M4) showed good linearity in the range of 0.1~50 ng/mL with R^2^ > 0.999; and BPC-157 (2–15) free acid (M8) had a linear range of 0.2~50 ng/mL with R^2^ > 0.999 (Table 3). Compared with a previous report [9], the detection method for BPC-157 in this study showed a wider linearity as well as for its major metabolites. Meanwhile, the RSD% for repeatability in the six replicates of urine samples varied from 1.39% to 3.91%, and the RE% ranged from −7.74% to 8.09% for all analytes at three concentration levels (1, 5, and 20 ng/mL), meeting the quantitative requirements and demonstrating the quantitative possibilities of the established method.

#### 2.3.3. Recovery, Matrix Interference, and Carryover

Although the previous study indicated that the recovery of BPC-157 on the WCX cartridge is poor (11.4% at 2 ng/mL) due to its acidic isoelectric point [9], the WCX SPE-based method developed here provided outstanding recovery for both BPC-157 and its metabolites. As shown in Table 3, all targets evaluated were extracted with a recovery rate higher than 90% at 2 ng/mL. In addition, the matrix had a slight ion suppression effect for all the targets (21.1~30.0%), and no carryover was observed after the analysis of a spiked sample at a concentration of 8 ng/mL.

#### 2.3.4. Reliability and Sample Extract Stability

According to the results of LOD (Table 3), all target analytes could be detected and identified in ten samples at 2 ng/mL with the operation performed on different days by different analysts, demonstrating that minor variations in the experimental conditions do not affect the results. Re-analyzing the batch of ten previously prepared urine samples at 2 ng/mL at time intervals of 48 h (autosampler, 10 °C) and 5 days (−20 °C) showed that all the targets can be detectable in 100% of the samples. Thus, the sample extract is considered stable under the instrument autosampler (10 °C) for 48 h and −20 °C for 5 days.

## 3. Materials and Methods

### 3.1. Chemicals and Reagents

Mixed-gender human liver microsomes (10-Donor Pool, total protein concentration of 20 mg/mL) and mixed-gender human skin S9 (3-Donor Pool, total protein concentration of 1.89 mg/mL) were purchased from BioreclamationIVT (Westbury, NY, USA). Phosphate buffered saline (PBS, pH 7.2~7.4) was obtained from Sangon Biotech Co., Ltd. (Shanghai, China). BPC-157 (HPLC purity > 99%) and (Deamino-cysl, val4, D-arg8)-Vasopressin (DCVDV, internal standard, ISTD) were purchased from Alta Scientific Co., Ltd. (Tianjin, China). ^13^C_6_,^15^N_2_-BPC-157 (labeled BPC-157, HPLC purity > 98%) was purchased from Synpeptide Co., Ltd. (Nanjing, China). BPC-157 (2–15) free acid, BPC-157 (1–13) free acid, BPC-157 (2–13) free acid, BPC-157 (1–12) free acid, BPC-157 (3–15) free acid, BPC-157 (1–8) free acid, and BPC-157 (2–8) free acid were synthesized by Alta Scientific Co., Ltd. (Tianjin, China). MgCl_2_ was from Beyotime Biotechnology (Shanghai, China). Nicotinamide adenine dinucleotide phosphate (NADPH) was purchased from Vetec (Shanghai, China). Acetonitrile (ACN), methanol (MeOH), and formic acid (FA) were of LC-MS grade and were obtained from Thermo Fisher Scientific (San Jose, CA, USA). Milli-Q purified water (MerckMillipore, Vimodrone, Milan, Italy) was used for sample preparation, reference material dilution, and LC mobile phase preparation. Mixed-mode weak cation exchange cartridges, Oasis WCX (30 mg, 1 cc), were purchased from Waters (Milford, MA, USA). Protein LoBindtubes (1.5, 2, and 5 mL) were obtained from Eppendorf (Hamburg, Germany). A positive pressure SPE system “Biotage Pressure+48” was purchased from Biotage Trading Co., Ltd. (Uppsala, Sweden).

### 3.2. Standard Solution Preparation

BPC-157 and labeled BPC-157 were reconstituted in PBS at a concentration of 0.4 mmol/L, respectively, and stored at −80 °C for long-term use. MgCl_2_ solution (1 M) was diluted with PBS at a concentration of 0.1 mol/L. NADPH solution, which needed to be prepared immediately before use, was reconstituted in PBS at a concentration of 0.1 mol/L.

Standard and ISTD stock solutions were prepared with the solvent mixture of H_2_O/ACN/FA (49.5/49.5/1, *v*/*v*/*v*) in LoBind tubes and stored at −80 °C. A series of mixed standard solutions (including BPC-157 and the five metabolites) at levels of 2, 5, 10, 20, 50, 100, 200, 500, 1000, 2000, and 5000 ng/mL were prepared for method validation. The working solution of ISTD was prepared at a concentration of 1 μg/mL. Blank urine samples were obtained from healthy volunteers (both male and female) without any known medication administration.

### 3.3. In Vitro Metabolic Incubation of BPC-157 and Labeled BPC-157

The in vitro metabolism study was performed with mixed-gender human liver microsomes (the final protein concentration was 1 mg/mL) and human skin S9 (the final protein concentration was 0.189 mg/mL). In detail, 10 µL of the standard solution of BPC-157 or labeled BPC-157 was added to the human liver microsomes incubation system (consisting of 160 µL of PBS solution, 10 µL of liver microsomes solution, 10 µL of MgCl_2_ solution and 10 µL of NADPH solution) and the human skin S9 incubation system (consisting of 180 µL of PBS solution and 10 µL of human skin S9 solution), respectively. Then, the incubation system was vortexed for 30 s followed by incubation at 37 °C for 2 h. Afterward, the sample solution was fortified with 200 µL of frozen acetonitrile and centrifuged for 10 min at 12,000 rpm. Then, 100 µL of the supernatant was diluted with 400 µL of water and transferred into HPLC vials for analysis by UHPLC-HRMS. In all series of incubation experiments, enzyme blank (without human liver microsomes and NADPH/human skin S9) and substrate blank (without BPC-157/labeled BPC-157) samples were prepared to allow for differentiating metabolic from incubation artifact reactions.

### 3.4. Urine Sample Preparation

In total, 15 µL of ISTD solution and 5 µL of FA were added to 1.5 mL of urine sample successively. Then, samples were vortexed automatically for 3 min and centrifuged for 5 min at 3500 rpm. The extraction of target analytes in urine samples was performed on a positive-pressure SPE system. First of all, WCX SPE cartridges were activated with 1 mL of MeOH and balanced with 1 mL of water. Then, urine samples (1 mL) were loaded onto the cartridges and washed with 1 mL of water. The target analytes were eluted with 1 mL of elution reagent (75% ACN and 5% FA in water) directly into the LoBind tubes. The eluates were evaporated to dryness under a nitrogen stream at 38 °C. Then, 100 μL of water with 10% ACN and 0.2% FA was added to the tube for reconstitution. Samples were vortexed and centrifuged for 10 min at 12,000 rpm, and the supernatants were transferred into vials for analysis.

### 3.5. Instrument Parameters and Data Processing

All LC-MS experiments were performed on a Vanquish Flex system coupled with an Orbitrap Explories 480 mass spectrometer (ThermoFisher Scientific, Bremen, Germany). LC separations were carried out on a reversed-phase BEH C18 column (100 mm × 2.1 mm, 1.7 µm; Waters, Milford, CT, USA) at 30 °C with a flow rate of 0.25 mL/min. The mobile phases were ultrapurified water (eluent A) and ACN (eluent B), both containing 0.1% of FA. The gradient elution profile was as follows: 0–1 min, 3% B; 1–10 min, 3% to 50% B; 10–13 min, 50% to 95% B; 13–15 min, 95% B; 15–15.1 min, 95% to 3% B; 15.1–20 min, 3% B. Samples were stored at 10 °C in the autosampler prior to analysis and the injection volume was fixed at 5 μL. The mass spectrometer was operated with a heated electrospray ionization (HESI) source in positive ion mode. The spray voltage was 3.8 kV, the capillary temperature was 320 °C, and the auxiliary (AUX) gas heater temperature was 380 °C. The nitrogen sheath and AUX gas flow rates were set to 40 and 10, respectively. For the full scan mode, the resolution was set at 60,000 (*m*/*z* 200), the mass range was 100 to 1500 *m*/*z*, and the S-lens radio frequency (RF) level was set to 40. For parallel reaction monitoring (PRM) mode, the resolution was set at 15,000 (*m*/*z* 200), and the isolation window was 2 *m*/*z*. The optimized normalized collision energies (NCEs) for each analyte are summarized in Table 4.

The instrument was operated using Xcalibur 4.5.474.0, and the data were processed using TraceFinder 5.1 (Thermo Scientific, Waltham, MA, USA). Compound Discoverer 3.1 (Thermo Scientific, USA) was used for the ion extraction with the following key parameters: (1) ion peak signal intensity ≥ 50,000; and (2) ions detected as [M+H]^+^, [M+2H]^2+^, and [M+3H]^3+^. For automatic ^13^C_0_, ^15^N_0_-^13^C_6_, ^15^N_2_ *m*/*z* pair picking, the resulting molecular features were exported into Excel (Microsoft, Redmond, WA, USA) and then analyzed using a custom program implemented in Python 3.10.

### 3.6. Method Validation

The analytical method developed for qualitative and quantitative analysis of BPC-157 and its main metabolites in human urine using WCX-SPE combined with UHPLC-HRMS was validated according to WADA technical documents.

#### 3.6.1. Selectivity and LOD

Twenty blank urine samples (males and females) were analyzed in two batches to evaluate the ability of the method to differentiate target analytes from endogenous matrix interferences in the sample, and interference at the stated RT shall not appear in all samples for the product ions shown in Table 4. Ten urine samples (males and females) fortified with the analytes at desired concentrations (0.02, 0.1, 0.2, 0.5, 1, and 2 ng/mL) were analyzed by two analysts over different days for the estimation of LOD, and an S/N of the product ion peak larger than 3 was considered as detected. The LOD is defined as the concentration where a 95% detection rate was reached according to a sigmoid fitting function (R version 4.1.0).

#### 3.6.2. Linearity, Repeatability, and Accuracy

Linearity was assessed by analyzing urine samples fortified to final concentrations of 0.02, 0.05, 0.1, 0.2, 0.5, 1, 2, 5, 10, 20, and 50 ng/mL for all target analytes. The concentrations were plotted against the peak area of target analytes, and the regression coefficient was calculated. Six replicates of urine samples spiked at low, middle, and high three different concentration levels (1, 5, and 20 ng/mL) were prepared and assayed for the evaluation of repeatability and accuracy. Repeatability was evaluated by calculating the RSD% of the peak area of the target analytes in six replicates. Accuracy was calculated based on the established calibration curves and expressed as the relative error in percent (RE%), RE% = (measured concentration value—actual concentration value)/actual concentration value * 100(%).

#### 3.6.3. Recovery, Matrix Interference, and Carryover

The recovery of the method was evaluated at a concentration of 2 ng/mL. One set of ten urine samples was fortified with target analytes prior to extraction and compared to another set of the same ten urine samples fortified with target analytes after extraction. The ratio of the peak areas in two sets for each analyte was calculated. In addition, ten blank urines and ten aliquots of water were prepared before adding the standard solution to a final concentration of 2 ng/mL to evaluate the interferences due to co-eluting matrix components. The mean peak area of ten spiked urines was compared to the mean peak area of spiked water for each analyte. The risk of carryover was investigated by analyzing one urine sample spiked at 8 ng/mL, followed by consecutive analysis of two extracted blank samples, and the results were evaluated with regard to the presence of target signals in the blank sample.

#### 3.6.4. Reliability and Sample Extract Stability

The reliability was assessed to ensure the production of consistent results in the case of routine variations in the experimental conditions, such as different analysts and different days. Accordingly, ten representative urine samples spiked at 2 ng/mL were prepared and analyzed in two batches with five samples/batch by two analysts over different days. All target analytes must be detected and identified in ten samples. Sample extract stability was evaluated by re-analyzing the batch of ten previously prepared urine samples at 2 ng/mL at time intervals of 48 h (autosampler, 10 °C) and 5 days (−20 °C). The loss of detection response was allowed, while the detection rates for all analytes were required to be 100%.

## 4. Conclusions

In summary, a stable isotope labeling-based nontargeted strategy combined with UHPLC-HRMS was first proposed for the metabolism analysis of small-molecule doping agents and demonstrated via its application in the deeper in vitro metabolic profiling of a new peptide doping BPC-157. Based on the similar chromatographic behavior and fixed mass differences of isotope pairs, a complete workflow including automatic ^13^C_0_,^15^N_0_-^13^C_6_,^15^N_2_ *m*/*z* pair picking was developed, which realized the rapid and effective screening of targets from a large amount of mass spectral information. As a result, extensive metabolism was observed in the human liver microsomes and human skin S9 incubation systems with an obvious difference, and one metabolite produced by a novel metabolic pathway plus eight metabolites produced by the conventional amide-bond breaking metabolic pathway was discovered. The five main metabolites were identified with the aid of synthetic RMs. Furthermore, a sensitive and specific method for the detection of BPC-157 and the five main metabolites in the human urine samples was developed and validated in accordance with the criteria of WADA for the first time, which was also capable of quantitative analysis for relatively high recovery, ideal accuracy, and precision. To our knowledge, the novel metabolic pathway was first discovered for BPC-157 and can possibly provide new insights into the biotransformation of not only BPC-157 but also other small peptides. The in vitro metabolic profile could provide a further reference for the in vivo metabolism of BPC-157 and improved analytical targets for doping control.

## Figures and Tables

**Table 1:** The five possible molecular formulas of M9.

No.	Molecular Formula	Molecular Weight	Delta ppm
1	C_67_H_104_O_27_N_12_	1508.71339	−0.015
2	C_64_H_96_O_21_N_22_	1508.71204	0.787
3	C_68_H_100_O_23_N_16_	1508.71472	−0.990
4	C_63_H_100_O_25_N_18_	1508.71070	1.673
5	C_69_H_96_O_19_N_20_	1508.71606	1.876

**Table 2:** Nine different metabolic products of BPC-157 in two in vitro incubation models.

No.	Compound	Sequence	Molecular Weight	RT (min)	*m*/*z* (+2)	Peak Height (Human Skin S9)	Peak Height (Human Liver Microsomes)
1	BPC-157 (parent)	GEPPPGKPADDAGLV	1419.53	7.45	710.35936	4.4 × 10^8^	4.5 × 10^8^
2	BPC-157 (2–8) free acid (M1)	EPPPGKP	720.81	5.01	361.19760	9.5 × 10^5^	8.3 × 10^5^
3	BPC-157 (1–8) free acid (M2)	GEPPPGKP	777.86	5.14	389.70833	9.7 × 10^5^	7.2 × 10^6^
4	BPC-157 (2–13) free acid (M3)	EPPPGKPADDAG	1150.19	5.16	575.77238	4.4 × 10^5^	ND
5	BPC-157 (1–13) free acid (M4)	GEPPPGKPADDAG	1207.24	5.23	604.28312	5.4 × 10^6^	5.9 × 10^4^
6	BPC-157 (2–14) free acid (M5)	EPPPGKPADDAGL	1263.35	6.90	632.31442	ND	5.2 × 10^4^
7	BPC-157 (1–14) free acid (M6)	GEPPPGKPADDAGL	1320.40	6.91	660.82515	1.8 × 10^5^	2.0 × 10^6^
8	BPC-157 (6–15) free acid (M7)	GKPADDAGLV	942.02	7.15	471.74818	ND	8.9 × 10^5^
9	BPC-157 (2–15) free acid (M8)	EPPPGKPADDAGLV	1362.48	7.46	681.84862	3.7 × 10^7^	9.4 × 10^6^
10	M9	unknown	see Table 1	8.03	755.36389 ([M+2H]^2+^); 763.87708 ([[M+H+NH_4_]^2+^])	1.0 × 10^6^; 5.2 × 10^6^	ND

**Table 3:** Prominent results of the method validation.

No.	Compound	LOD (ng/mL)	Linearity (ng/mL)	Repeatability (*n* = 6, RSD%) (ng/mL)			Accuracy (RE%) (ng/mL)			Recovery (%)	Matrix Effect (%)
				1	5	20	1	5	20		
1	BPC-157	0.01	0.02–50	1.53	1.93	2.17	−2.96	1.99	8.09	93.73	75.0
2	BPC-157 (2–8) free acid (M1)	0.07	0.1–50	2.16	3.91	1.89	−0.15	−2.94	−0.63	109.48	74.0
3	BPC-157 (1–8) free acid (M2)	0.03	0.05–50	2.87	3.62	2.34	−3.91	−7.74	1.71	123.01	70.0
4	BPC-157 (2–13) free acid (M3)	0.07	0.1–50	3.55	2.33	2.5	3.87	0.03	3.02	116.28	75.4
5	BPC-157 (1–13) free acid (M4)	0.07	0.1–50	2.63	3.15	2.5	3.92	3.84	4.98	110.10	73.2
6	BPC-157 (2–15) free acid (M8)	0.11	0.2–20	2.2	1.77	1.39	5.92	0.81	7.81	93.79	78.9

**Table 4:** LC and MS parameters of the target analytes.

No.	Compound	Molecular Formula	RT (min)	Charge	Precursor Ion ^1^	Product Ion ^1,2^	NCE
1	BPC-157	C_62_H_98_N_16_O_22_	7.45	2	710.35936	617.32733 651.81873 558.78784	30
2	BPC-157 (2–8) free acid (M1)	C_33_H_52_N_8_O_10_	5.01	2	361.1976	352.19183 248.14960 398.23975	15
3	BPC-157 (1–8) free acid (M2)	C_35_H_55_N_9_O_11_	5.14	2	389.70833	296.67606 592.34412 187.07118	15
4	BPC-157 (2–13) free acid (M3)	C_49_H_75_N_13_O_19_	5.16	2	575.77238	462.72519 827.38922 924.44135	20
5	BPC-157 (1–13) free acid (M4)	C_51_H_78_N_14_O_20_	5.23	2	604.28312	511.25076 511.75076 462.72427	15
6	BPC-157 (2–15) free acid (M8)	C_60_H_95_N_15_O_21_	7.46	2	681.84862	681.84860 1114.51404 568.79956	15

^1^ Theoretical *m*/*z* values; ^2^ the first product ion was used for screening, and the three product ions were used for confirmation.

## Data Availability

Data will be made available on request.
